# Rap1 Is Essential for B-Cell Locomotion, Germinal Center Formation and Normal B-1a Cell Population

**DOI:** 10.3389/fimmu.2021.624419

**Published:** 2021-06-01

**Authors:** Sayaka Ishihara, Tsuyoshi Sato, Risa Sugioka, Ryota Miwa, Haruka Saito, Ryota Sato, Hidehiro Fukuyama, Akihiko Nakajima, Satoshi Sawai, Ai Kotani, Koko Katagiri

**Affiliations:** ^1^ Department of Biosciences, School of Science, Kitasato University, Sagamihara, Japan; ^2^ Laboratory of Lymphocyte Differentiation, RIKEN Center for Integrative Medical Sciences (IMS), Yokohama, Japan; ^3^ Department of Basic Science, Graduate School of Arts and Sciences, University of Tokyo, Tokyo, Japan; ^4^ Department of Hematological Malignancy, Institute of Medical Science, Tokai University, Isehara, Japan

**Keywords:** B cells, Rap1, germinal center, B-1a, chemotactic factor

## Abstract

Integrin regulation by Rap1 is indispensable for lymphocyte recirculation. In mice having B-cell-specific *Rap1a/b* double knockouts (DKO), the number of B cells in lymph nodes decreased to approximately 4% of that of control mice, and B cells were present in the spleen and blood. Upon the immunization with NP-CGG, DKO mice demonstrated the defective GC formation in the spleen, and the reduced NP-specific antibody production. *In vitro*, Rap1 deficiency impaired the movement of activated B cells along the gradients of chemoattractants known to be critical for their localization in the follicles. Furthermore, B-1a cells were almost completely absent in the peritoneal cavity, spleen and blood of adult DKO mice, and the number of B-cell progenitor/precursor (B-p) were reduced in neonatal and fetal livers. However, DKO B-ps normally proliferated, and differentiated into IgM^+^ cells in the presence of IL-7. CXCL12-dependent migration of B-ps on the VCAM-1 was severely impaired by Rap1 deficiency. Immunostaining study of fetal livers revealed defects in the co-localization of DKO B-ps and IL-7-producing stromal cells. This study proposes that the profound effects of Rap1-deficiency on humoral responses and B-1a cell generation may be due to or in part caused by impairments of the chemoattractant-dependent positioning and the contact with stromal cells.

## Introduction

The small GTPase Ras-related protein1(Rap1) regulates multiple functions such as cell proliferation, differentiation, and adhesion (1). Integrin-mediated regulation of lymphocyte adhesion and migration is an integral process at each step of immunosurveillance (2, 3). Rap1 is rapidly activated by chemotactic factors, induces the adhesiveness of integrins to their ligands, and promotes cell polarity, which in turn facilitates the directional movement of T and B lymphocytes and their interaction with antigen presenting cells (APC) and endothelial cells (4–8). We demonstrated that Rap1-RAPL-Mst1 pathway is essential for LFA-1-dependent arrest of T and B cells on the high endothelial cells (HEV), which is critical step to home into peripheral lymph nodes (4, 9, 10).

Rap1 has 2 isoforms, Rap1a and Rap1b, which share 95% amino acid identity. Previous papers (11, 12) demonstrated the critical role of Rap1b in B-cell trafficking and differentiation using Rap1b null mice, because Rap1b is the dominant isoform of Rap1 in lymphoid cells. Rap1b-deficiency leads to reduce B-cell population in lymph nodes (LNs), and impairs the development of marginal zone B cells (11, 12), but does not affect B-1 cell development (12). There is some difference in the effects of Rap1b-deficiency on early bone marrow development and humoral responses between these papers (11, 12). It is necessary to explore the exact roles of Rap1 in B-cell development using the mice having B-cell specific double knockout of Rap1a and Rap1b.

During immune responses, B cells undergo a series of migratory events and the structure of B-cell follicles dramatically changes to facilitate the efficient production of antibodies. Epstein-Barr virus-induced molecule 2 (EBI2; also known as G-protein- coupled receptor [GPR]183) and its ligand, 7α,25-dihydroxycholesterol(7α,25-OHC), as well as C-C chemokine receptor (CCR)7, chemokine (C-C motif) ligand (CCL)21, C-X-C chemokine receptor (CXCR)5, and chemokine (C-X-C) motif ligand (CXCL)13, direct the migration of activated B cells in the follicles, and guide them to the appropriate microenvironments (13, 14). Finally, activated B cells proliferate and form germinal centers (GCs) in the center of follicles.

GCs require proper compartmentalization for an optimal immune response, and are organized into two major zones: dark and light zones (15). The dark zone contains large centroblasts that are rapidly proliferating and undergoing somatic mutation (15). It has been suggested that these cells give rise to centrocytes in the light zone that compete for antigen binding on follicular dendritic cells (FDCs) and are then dependent on receiving signals from helper T cells to survive and differentiate (16). GC organization depends on sorting of centroblasts by CXCR4 into the dark zone, because centroblasts have high CXCR4 expression and migrate toward the CXCL12, which is more abundant in the dark zone than in the light zone (17). In contrast, CXCR5 is unnecessary for the segregation of dark and light zones (17).

Two main populations of B cells, referred to as B-1 and B-2 B cells, arise from distinct progenitors that emerge at different times during development (18, 19). B-2 cells generate specific antibodies against foreign antigens. B-1 cells are subdivided into B-1a and B-1b cells, of which many B-1a cells constitutively secrete natural immunoglobulin (Ig) M antibodies and participate in the antibody response against T-independent antigens, whereas B-1b cells can undergo clonal expansion and generate specific antibody to various antigens (20–22). B-2 cells are continually replenished from hematopoietic stem cells (HSCs) in the bone marrow. Although still a subject of investigation and some debate, in general terms, B-1b cells are derived both from the fetal liver and adult bone marrow B lymphopoiesis, whereas B-1a cells can differentiate from B-1 progenitors/precursors in the fetal and neonatal livers and are maintained throughout adult life by self-renewal (23–27). Previous paper demonstrates that neonatal spleen is required for B-1a cell maintenance (24). However, as the involvement of Rap1 in B-1 cell differentiation has only been examined using adult bone marrow (BM) cells (28, 29), the precise roles of Rap1 in B-1 cell development remain to be elucidated.

B-1 progenitors arise in the embryo before B-2 progenitors, and decline by young adulthood (19). B-1 cells emerge in a distinct neonatal wave of development within 2 weeks after birth (30). On embryonic day (E) 12.5 in mice, the fetal liver becomes a major B-1 lymphopoietic and myelopoietic organ where progenitors and precursors develop progressively with time until mature B-1 cells appear between E18.5 and E19.5 (birth) (30). Vascular cell adhesion molecule (VCAM)-1-positive stromal cells support hematopoiesis, because very late antigen-4 (VLA-4), the ligand of VCAM-1, is expressed on early hematopoietic cells and plays important roles in hematopoiesis (31). Activated leukocytes cell adhesion molecule (ALCAM) is another marker expressed by these stromal cells. ALCAM^high^ fetal liver cells produce IL-7 and chemokines such as CXCL12, and B-cell progenitor/precursors (B-p) including multiple stages (pro-B to pre-B) of the differentiation are chemoattracted to ALCAM^high^ cells, and proliferate and differentiate when in contact with them (23, 30, 32).

In this study, using mice harboring B-cell specific knockouts of *Rap1a* and *Rap1b* (DKO mice), we demonstrate that Rap1 is not only indispensable in B-cell population of peripheral lymph nodes, but is also a key factor in B-cell locomotion and may be indirectly involved in T cell-dependent antibody production and B-1a cell generation.

## Materials and Methods

### Mice

All animal experiments were carried out in accordance with Regulations for the Care and Use of Laboratory Animals in Kitasato University, and the protocols used in this study were ethically approved by the Institutional Animal Care and Use Committee for Kitasato University. *Rap*1a^f/f^ mice containing floxed exons 2–3 of *Rap1a*, *Rap1b* ^f/f^ mice containing floxed exon 1 of *Rap1b* on C57BL/6J background were maintained under specific pathogen–free conditions (33). Those mice were crossed with mb-1-Cre mice, yielding mice with B cell–specific deletion of *Rap1a/b* (33). Fetal liver was obtained from timed mating of WT or DKO mice. The embryonic stage was designed relative to embryonic day (E) 0.5, the day of plug formation. B cells were purified from the spleens of WT and DKO mice using B cell isolation kit, mouse (Miltenyi Biotec).

### Antibodies and Reagents

Fluorescence-conjugated anti-mouse CD45 allophycocyanin (APC), phycoerythrin(PE) Cy7, Brilliant Violet™ 421, CD3 FITC, B220 PE, APC, Brilliant Violet™ 711, CD19 FITC, PE, APC, IgM FITC, PECy7, APC, IgD PE, CD21 FITC, CD23 PE, CD24 PECy7, CD43 PE, CD5 PECy7, CD62L FITC, GL-7 APC, CD93 APC, LFA-1 PE, VLA-4 APC, CXCR4 APC, CXCR5 APC, CD35 FITC and IL-7R APC, CD4 FITC, CD8 FITC, CD11b FITC, Gr-1 FITC, NK1.1 FITC, TER119 FITC (e-bioscience or BioLegend), anti-ALCAM (R&D Systems), anti-Rap1 (BD Biosciences), Extracellular signal-regulated kinase (ERK), p-ERK, Akt kinase (Akt), p-Akt, β-actin and peroxidase-conjugated goat anti-mouse IgG (Cell Signaling) were used for flow cytometry, immunostaining and immunoblotting. Mouse CXCL13, CXCL12 and 7α,25-OHC were purchased from R&D Systems and Sigma. NIP-APC (4-hydroxy-3-iodo-5- nitrophenylacetate-allophycocyanin) was generated in-house (34).

### Flow Cytometry and Cell Sorting

Immunofluorescence flow cytometry was performed as described previously (9). For mAbs staining, cells were washed with staining buffer (1% FBS in HBSS), resuspended in 50 μl of the same buffer, pre-incubated with purified anti-mouse CD16/32 (Biolegend) for 10 min, and incubated for 30 min at 4°C with each fluorescence-conjugated mAb or isotype control matched with primary antibody. Zombie NIR™ dye (Biolegend) was used to assess live or dead status of cells. The samples were measured using a Gallios flow cytometry or CytoFLEX (Beckman Coulter). Doublets were distinguished from single cells by plotting FSC height vs FCS area. For the isolation of B-ps, fetal liver cells of E15-15.5 mice were immunostained as above, and CD45^+^ LIN^-^ (CD3^-^, CD4^-^, CD8^-^, CD11b^-^, Gr-1^-^, NK1.1^-^, TER119^-^) CD19^+^B220^+^CD93^+^ IgM^-^cells were sorted using a Moflo XDP instrument (Beckman Coulter). The purity of the sorted populations constituted more than 95% as determined by a presorted sample run in parallel. Data were analyzed in Kaluza analysis version 2.1 (Beckman Coulter).

### Immunoblot Analysis

B cells were lysed in buffer (1% Nonidet P-40, 150 mM NaCl, 25 mM Tris-HCl [pH 7.4], 10% glycerol, 2 mM MgCl_2_, 1 mM phenylmethylsulfonylfluoride, 1 mM leupeptin, and 0.1 mM aprotinin). Cell lysates were subjected to immunoblotting (35).

### Histological Examination

Preparation of frozen sections of the spleens, LNs and fetal livers from WT and DKO mice were performed as described previously (33). Sections were blocked for 1h at 20°C with PBS containing 10% goat serum and 0.1% Triton X-100 and incubated overnight at 4°C with the indicated antibodies. Nuclei were stained with SlowFade Gold antifade reagent with DAPI (invitrogen). Sections were examined on TCS SP8 (Leica).

### Homing Assay

Purified B cells were labeled with 1 µM 5, 6-carboxyfluorescein diacetate (CFSE, Invitrogen) and 10 µM (5-(and-6)) ((4-chloromethyl) benzoyl) amino) tetramethylrhodamine) (CMTMR, Invitrogen). An equal number of labeled control and Rap1-deficient B cells (1–5 × 10^6^) was injected intravenously into a normal C57BL/6 mouse. After 1 hr, inguinal and axillary LN cells, splenocytes and peripheral blood mononuclear cells were analyzed by flow cytometry (4).

### Lymphocyte Migration on ICAM-1 and VCAM-1

Migration on ICAM-1 or VCAM-1 was performed as previously described using a ΔT dish (Bioptechs Inc.) with immobilized recombinant mouse ICAM-1Fc (0.2 µg/ml) or mouse VCAM-1Fc (0.2 µg/ml) (36, 37). A total of 1 × 10^6^ cells were loaded onto the ICAM-1 or VCAM-1-coated dish. Phase-contrast images were obtained using an Olympus Plan Fluor DL 10 ×/0.3NA objective every 15 sec for 10-15 min at 37°C using a heated stage for ΔT dishes (Bioptechs Inc.). The frame-by-frame displacements and lymphocyte velocities were calculated by automatically tracking individual cells using MetaMorph software (Molecular Devices). In each field, 30 randomly selected cells were manually tracked to measure the median velocity and displacement from the starting point.

### Chamber Fabrication and Assay for Chemotaxis Towards Chemokine Gradient Using Chambers

A micro-chamber for the chemotaxis assay was fabricated following a photolithography process described earlier (38–40). In brief, polydimethylsiloxane (PDMS; Sylgard 184 Silicone Elastomer Kit, Dow Corning) solution with a mixing ratio of 10:1 (base: curing agent) was poured on a 50 µm-thick SU8-mold (SU-8 3050; MicroChem) and was cured for 1 hr at 75°C. The PDMS sheet was then peeled off from the mold. Inlets for chemoattractant and cell-loading were opened with a 1.5-mm or 2-mm diameter biopsy punch (BP-15F, BP-20F; Kai industries), respectively. The fabricated PDMS was cut using a stainless steel corer (BSV01; TKG) to form a round 10-mm diameter disc. A glass-bottom dish (P35G-0-14-C; MatTek) was treated with O_2_ plasma for 10 min to clean the glass surface using a plasma etcher (FA-1; Samco). The dish and the PDMS disc were treated with an O_2_ plasma for an additional 5 sec, attached together by hands and immediately heated on a hot plate for 3 min at 80°C for permanent bonding.

Custom-made migration chambers were coated with mouse VCAM-1 Fc (0.2 µg/ml), and overlaid with 30 µl of 200 nM CXCL12 or 200 nM 7α, 25-OHC with 10 µl of 0.4 µg/ml Alexa Fluor 594 dye (Thermo Fisher Scientific). The dimensions of the chamber are 500 µm wide, 50 µm high and 1mm long. B cells from the spleens of WT and DKO mice were cultured with 5µg/ml of anti-IgM F(ab’)_2_ and 2ng/ml of IL-4 for 2 days. 10 µl of activated B-cell suspension (5x10^5^ cells) was casted into the chamber, and observed at 37°C for 180 min *via* time-lapse video microscopy. Cells were tracked using MetaMorph software.

### [^3^H]Thymidine Incorporation Assay

Purified B cells or B-ps were plated into 96-well plates in triplicates and stimulated with 2 and 10 µg/ml goat-mouse IgM F(ab́)_2_ or 5 ng/ml IL-7 for 24-48 h. A total of 1mCi [^3^H] thymidine (GE health Life Science) was added 6h before harvest. Labeled DNA from cells was collected on GSC filters (Whatman), and the radioactivity was measured in a scintillation counter.

### Immunization With NP-CGG (Nitrophenyl-Chicken Gamma Globulin)

WT and DKO mice were intrapenitoneally injected with 100-200 µg of NP-CGG emulsified in complete Freund’s adjuvant (Sigma-Aldrich). The spleens from day 14 and 20 immunized WT and DKO mice were frozen, and the crystal sections were stained with the indicated antibodies. Sera were diluted and analyzed by ELISA using microplates coated with NP_30_ or NP_11_-BSA (4-Hydroxy-3- nitrophenylacetyl hapten conjugated to Bovine Serum Albumin) and NP-specific Ig isotypes using Clonotyping System (Southern Biotechnology Association).

### Flow-Adhesion Assay

The human endothelial cell line LS12 was introduced with mouse ICAM-1 (40). They were cultured on fibronectin-coated disk and pretreated with TNFα. These disks were incubated with or without CXCL13 and placed in the flow chamber (FCS2; Bioptechs). Shear flow was generated using an automated syringe pump (Harvard Apparatus). B cells were infused in pre-warm RPMI1640 medium were infused into the flow chamber at 2 dyne cm^-2^ at 37°C. Images were recorded at 3.3-ms. Frame-by-frame displacements and velocities of B cell movements were calculated by automatically tracking individual cells using the MetaMorph software (Molecular Devices). Interaction with LS12 cell was categorized depending on dwell time: rolling; transient adhesion (0.5-10s); and stable arrest (more than 10s). the frequencies of cells exhibiting rolling, transient and stable arrest per minute are shown.

### Detachment Assay

CXCL13-stimulated B cells adhesion assays were performed using a temperature-controlled parallel flow chamber (FCS2, Bioptechs Inc.), with immobilized recombinant ICAM-1Fc (36). Purified B cells were incubated with 100nM CXCL13 for 10 min and then shear stress was applied for 1min at 2 dyne/cm^2^.

### Pull-Down Assay

Rap1-GTP was pulled down with a GST (Glutathione S-transferase)-RBD (Ras-binding domain) of Ral guanine nucleotide dissociation inhibitor (GDS) fusion protein. Briefly, 10^7^ cells were lysed in ice-cold lysis buffer [1% Triton X-100, 50 mM Tris-HCl (pH7.5), 100 mM NaCl_2_, 10mM MgCl_2_, 1mM phenylmethylsulfonyl fluoride, 1mM leupeptin] and incubated for 1h at 4°C with GST-fusion proteins coupled to glutathione agarose beads. The beads were washed three times with lysis buffer and subjected to western blot analysis using anti-Rap1 antibody.

### Statistical Analysis

Statistical analysis was performed using two-tailed Student’s t-test. *P* values less than 0.05 were considered significant.

## Results

### The Effects of Rap1 Deficiency on B Cell Distribution

**Figure 1:**
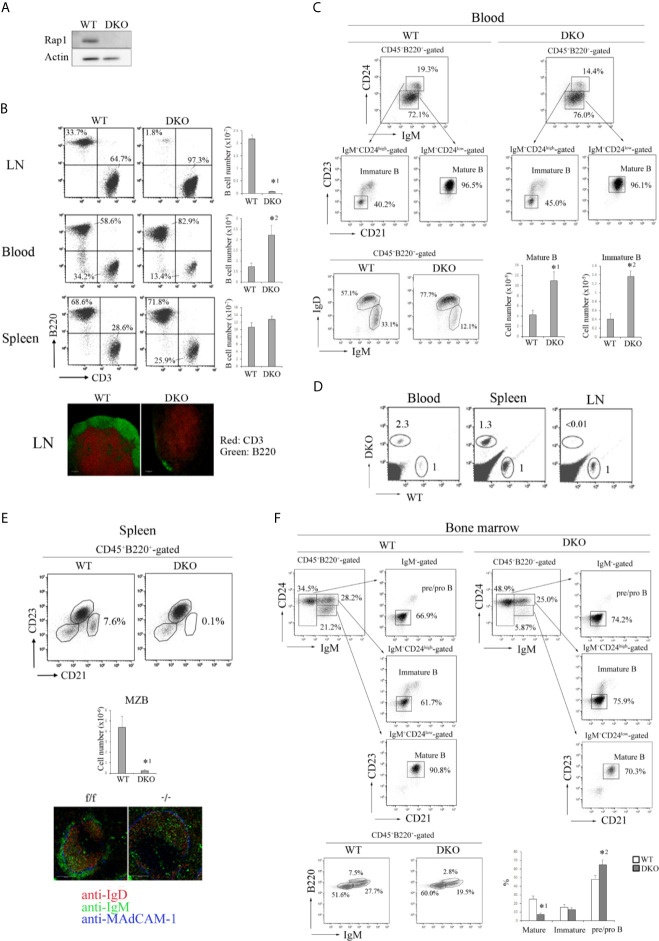
Effects of Rap1-deficiency on the distribution of B cells. **(A)** Expression of Rap1 in WT and DKO B cells. Actin is a loading control. **(B)** (Top, left) B220 and CD3 profiles of CD45^+^-gated cells from the LN, blood and spleen of WT and DKO mice. The numbers indicate the percentages of B220^+^ B cells. (Top, right) T and B cell numbers in the LN, blood and spleen. (*n*=5-7). The mean values and standard errors are shown. *^1^ *p* < 0.001, *^2^ *p*<0.008, compared with WT B cells. (Bottom) The sections of LNs from WT and DKO were stained with anti-CD3 (red) and B220 (green). **(C)** (Top) IgM and CD24 profiles of CD45^+^B220^+^-gated blood cells, CD23 and CD21 profiles of IgM^+^CD24^high^ or IgM^+^CD24^low^ cells from WT and DKO mice. Numbers indicate the percentages of IgM^+^CD24^low^, IgM^+^CD24^high^, CD23^-^CD21^-^, CD23^+^CD21^+^ B cells. (Bottom, left) IgM and IgD profiles of CD45^+^B220^+^-gated blood cells from WT and DKO mice. Numbers indicate the percentages of IgM^+^ IgD^high^ and IgM^+^ IgD^low^ B cells. (Bottom, right) The numbers of IgM^+^CD24^low^CD21^+^CD23^+^ mature and IgM^+^CD24^high^CD21^-^CD23^-^ immature B cells in the blood of WT and DKO mice (*n*=5). The mean values and standard errors are shown. *^1^ *p* < 0.009, *^2^ *p* < 0.001 compared with WT cells. **(D)** WT and DKO B cells were labeled with CFSE and CMTMR, respectively, and injected into normal mice. After 1 h, lymphocytes from the LN, spleen and blood of injected mice were analyzed. The numbers indicate the ratios of DKO cells relative to WT cells (adjusted to 1). **(E)** (Top) CD21 and CD23 profiles of CD45^+^B220^+^-gated splenocytes from WT and DKO mice. The numbers indicate the percentages of CD21^high^CD23^low^ marginal zone B cells to total CD45^+^B220^+^ cells. (Middle) The numbers of CD21^high^CD23^low^ marginal zone B cells in spleens of WT and DKO mice (*n*=5). The mean values and standard errors are shown. *p < 0.005, compared with WT cells. (Bottom) Spleen sections stained with anti-IgD (red), IgM (green) and MadCAM-1 (blue). Marginal zone B cells (IgM^+^ IgD^-^ cells) were located outside of MadCAM-1^+^ cells, and absent in the spleen of DKO mice. **(F)** (Top) IgM and CD24 profiles of CD45^+^B220^+^-gated bone marrow cells, CD23 and CD21 profiles of IgM^-^CD24^high^, IgM^+^CD24^high^ and IgM^+^CD24^low^ cells from WT and DKO mice. Numbers indicate the percentages of IgM^-^, IgM^+^CD24^high^, IgM^+^CD24^low^, CD23^-^CD21^-^, CD23^+^CD21^+^ B cells. (Bottom, left) IgM and B220 profiles of CD45^+^B220^+^-gated bone marrow cells from WT and DKO mice. Numbers indicate the percentages of IgM^-^B220^low^, IgM^+^B220^low^ and IgM^+^B220^high^ B cells. (Bottom, right) The numbers of IgM^+^CD24^low^CD21^+^CD23^+^ mature B cells, IgM^+^CD24^high^CD21^-^CD23^-^ immature B cells and IgM^-^CD21^-^CD23^-^ pre/pro B cells in the bone marrow of WT and DKO mice (*n*=6). The mean values and standard errors are shown. *^1^ *p* < 0.002, *^2^ *p* < 0.03 compared with WT cells.

To generate *Rap1a* and *Rap1b* conditional double-knockout (DKO) mice, mice carrying floxed *Rap1a* and *Rap1b* alleles (*Rap1* ^f/f^) were mated with mb-1-cre transgenic mice to specifically delete Rap1 in B cells. Western blot analysis confirmed that the Rap1 protein was not expressed in B cells derived from these mice (**Figure 1A**).

The number of B cells in the peripheral lymph nodes (LNs) of DKO mice diminished to less than 4% of that of wild-type (WT) mice, and Rap1-deficient B cells were present in the blood and spleen of DKO mice at 8-10 weeks of age (**Figures 1B, C**). WT and DKO B cells were differentially labeled and adoptively transferred into normal mice. The trafficking of DKO B cells to the peripheral LNs was reduced to less than 1% of that of WT B cells (**Figure 1D**). Marginal zone (MZ) B cells were absent in the spleen of DKO mice (**Figure 1E**). As expected, Rap1-deficient B cells exhibited severe impairment in attachment to immobilized intercellular adhesion molecule (ICAM-1) in the presence of CXCL13 (**Figure S1A** in **Supplementary Material**). The interaction of B cells with the high endothelial venules (HEV) was mediated by L-selectin-mediated rolling and chemokine-triggered integrin-dependent arrest. As previously reported (33), L-selectin-dependent rolling was increased in DKO B cells, but CXCL13 and lymphocyte function-associated antigen (LFA)-1-dependent stable arrest was completely abrogated by Rap1-deficiency (**Figure S1B** in **Supplementary Material**). Although CXCL13-dependent migration of DKO B cells on the ICAM-1 was significantly reduced (**Figure S1C** in **Supplementary Material**), the splenic architecture of B-cell follicles was not disordered in DKO mice (**Figures 1E** and **S1D** in **Supplementary Material**).

In addition to the increase in mature B cells (IgM^+^CD24^low^CD23^+^CD21^+^ or IgM^+^IgD^high^), immature B cells (IgM^+^CD24^high^CD23^-^CD21^-^ or IgM^+^IgD^low^) were also significantly elevated in the blood of DKO mice (**Figure 1C**), suggesting that Rap1-deficiency impairs the retention of immature B cells in the BM, which is mediated by CXCR4 and VLA-4 (41). The proportion of mature B cells (IgM^+^CD24^low^CD23^+^CD21^+^ or IgM^+^B220^high^) in the BM of DKO mice was significantly reduced, compared with that of WT mice (**Figure 1F**). Taken together, these results show that Rap1 plays a central role in B-cell homing into the LNs and BM, differentiation of MZ B cells in the spleen, and retention of immature B cells in the BM.

### Rap1 Is Involved in GC Formation Induced by NP-CGG Injection

**Figure 2:**
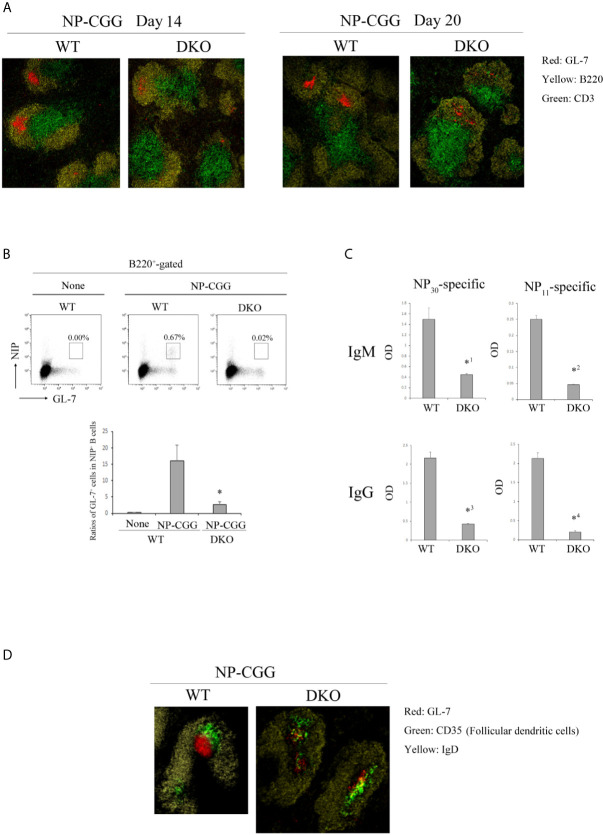
Decreased humoral response to NP-CGG in DKO mice. **(A)** Spleen sections of WT and DKO mice injected with NP-CGG (day14 and 20) were stained with anti-GL-7 (red), anti-B220 (yellow) and anti-CD3 (green). **(B)** (Top) Antigen-specific GC B cells (B220^+^, NIP^+^, GL-7^+^) in the spleens of WT and DKO mice uninjected or injected with NP-CGG were analyzed by flow cytometry. The numbers show the percentages of GL-7^+^ NIP^+^ cells in B cells. (Bottom) The percentages of GL-7^+^ cells in NIP^+^ B cells from the spleens of WT and DKO mice are shown (*n*=3). The mean values and standard errors are shown. *p < 0.02, compared with WT mice. **(C)** Anti-NP IgM and IgG titers in sera of WT and DKO mice injected with NP-CGG were measured by ELISA. NP_30_ and NP_11_ as hapten antigens were used for detecting low- and high-affinity anti-NP antibodies, respectively in triplicate. The mean values and standard errors are shown. *^1^ *p* < 0.009, *^2^ *p* < 0.001, *^3^ *p* < 0.001, *^4^ *p* < 0.001, compared with WT mice. **(D)** Spleen sections of WT and DKO mice injected with NP-CGG (day 20) were stained with anti-GL-7 (red), anti-IgD (yellow) and anti-CD35 (green).

To determine whether Rap1 is involved in humoral immunity by regulating the GC organization, we immunized the mice intraperitoneally with NP-CGG, and analyzed splenic GCs using immunostaining and flow cytometry. The GC cells demonstrated a segmented distribution and the proportion of GL-7^+^ NIP^+^ GC cells was significantly reduced in the spleen of injected DKO mice (**Figures 2A, B**). NP-specific antibody titers were determined by enzyme-linked immunosorbent assay (ELISA) using 96-well plates coated with NP_11_ or NP_30_-bovine serum antigen (BSA). Both high- and low- affinity antibodies bound to NP_30_-BSA, whereas only high-affinity antibodies bound to NP_11_-BSA. As shown in **Figure 2C**, injected DKO mice produced significantly lower amounts of both NP_11_- and NP_30_-specific IgM and IgG, compared to those of WT mice. These data indicate that Rap1 is required for the generation of GC cells.

GCs have two distinct zones, namely the dark and light zones, which are associated with important functional differences. We visualized the dark and light zones of GCs in the WT and DKO spleens by immunostaining. CD35^+^ FDCs were enriched in the light zone, and GL7^+^ GC cells accumulated densely in the dark zone of WT spleens (**Figure 2D**). However, CD35^+^ FDCs did not integrate at the distal pole of the light zone, and GL7^+^ GC cells scattered throughout the dark zone of DKO spleens (**Figure 2D**), suggesting that GC organization is also Rap1-dependent.

**Figure 3:**
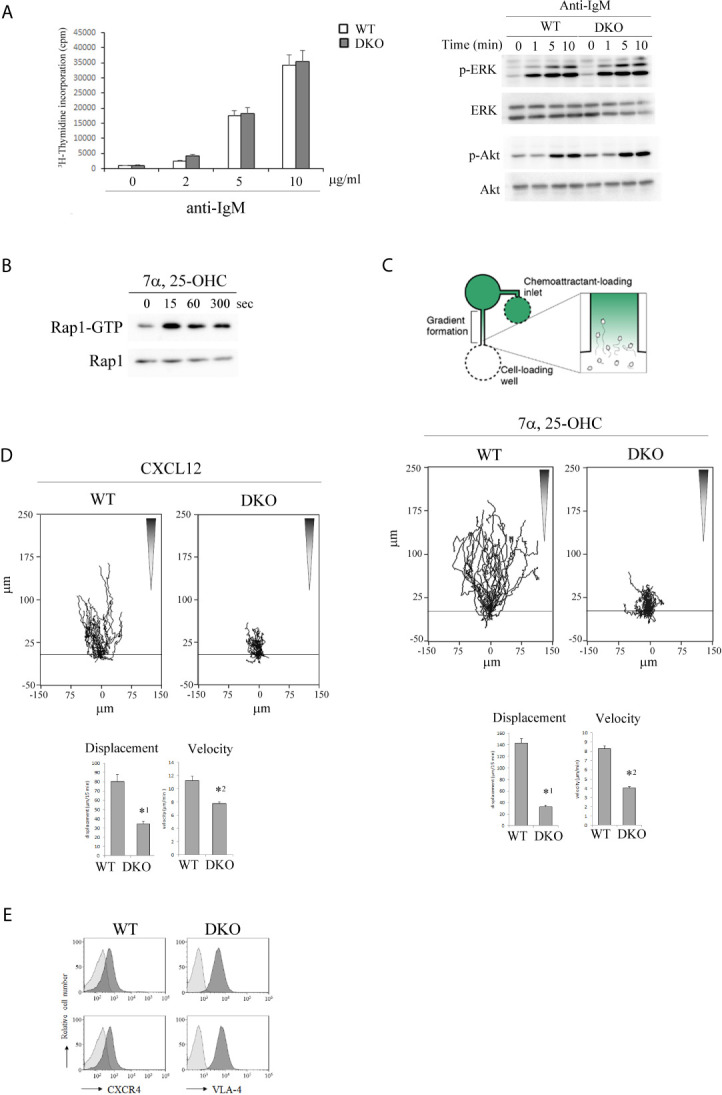
Rap1-deficiency causes defective locomotion of activated B cells along chemotactic factors. **(A)** (Left) [^3^H]-thymidine uptake by B cells. Primary B cells from the spleens of WT and DKO mice were unstimulated or stimulated with antigen receptor ligation by anti-IgM F(abʹ)_2_ at the indicated concentrations. [^3^H]-thymidine uptake was measured 48 hr after the stimulation in triplicate. The mean values and standard errors are shown. (Right) Phosphorylated and total ERK and Akt in stimulated WT and DKO B cells with 5µg/ml of anti-IgM F(abʹ)_2_ at the indicated times are shown. **(B)** WT B cells which were cultured in the presence of anti-IgM F(abʹ)_2_ and IL-4 for 2 days were stimulated with 200nM 7α,25-dihydrixycholesterol (7α,25-OHC) as indicated times. Cell lysates were pulled down with GST-Ral-GDS- RBD and immunoblotted with an anti-Rap1 antibody. **(C)** (Top) The experimental set up of scheme. B cells from the spleens of WT and DKO mice were stimulated with anti-IgM F(abʹ)_2_ and IL-4 for 2 days. Time lapse sequences of those activated B cells migrating toward the source of 7α,25-OHC were recorded. (Middle) Representative tracks of the activated WT and DKO B cells on the VCAM-1 in response to 7α,25-OHC gradient are shown. Each line represents a single cell track. (Bottom) Displacement and velocity of the activated WT and DKO B cells (*n*=30). The mean values and standard errors are shown. *^1^ *p* < 0.001, *^2^ *p* < 0.001, compared with WT cells. **(D)** (Top) Representative tracks of the activated WT and DKO B cells on the VCAM-1 in response to CXCL12 gradient are shown. Each line represents a single cell track. (Bottom) Displacement and velocity of the activated WT and DKO B cells (*n*=30). The mean values and standard errors are shown. *^1^ *p* < 0.001, *^2^ *p* < 0.001, compared with WT cells. **(E)** The expression of CXCR4 and VLA-4 on the activated WT and DKO B cells.

The proliferative response of Rap1-deficient B cells to anti-IgM F(ab’)_2_ was similar to that of WT B cells (**Figure 3A**). In addition, there were no differences in B cell antigen receptor (BCR)-mediated phosphorylation of ERK and Akt between WT and DKO B cells (**Figure 3A**). Therefore, the reduction in the number of antigen-specific GL-7^+^ GC cells in injected DKO mice was not due to the impaired BCR-mediated early signaling in response to antigens.

EBI2 and 7α,25-OHC were reported to play critical roles in positioning of antigen-activated B cells within lymphoid follicles, which is important for the initial burst of B cell proliferation and GC commitment (13, 14). Since chemokines activate Rap1 through Gi-protein coupled receptors (GPCRs) in T and B lymphocytes (36, 37, 40), we examined whether 7α,25-OHC activated Rap1 in activated B cells. As shown in **Figure 3B**, 7α,25-OHC continuously activated Rap1 during 5min from 15 sec in the activated B cells. We examined the effects of Rap1 on the locomotion of activated B cells along a 7α,25-OHC gradient. The locomotion of Rap1-deficient B cells on the VCAM-1 along the 7α,25-OHC gradient was significantly decreased (**Figure 3C**).

Previous papers have reported that the segregation of light and dark zones depends on the sorting of centroblasts by CXCR4 and CXCL12 into the dark zone (17). We examined the effects of Rap1 deficiency on the directed movement of activated B cells which sense the CXCL12 gradient. As shown in **Figure 3D**, the locomotion of activated Rap1-deficient B cells on the VCAM-1 along a CXCL12-gradient was significantly diminished, compared to that of activated WT B cells. There was no difference in the expression of CXCR4 and VLA-4 between WT and Rap1-deficient activated B cells (**Figure 3E**).

These data indicate that Rap1 is important for the GC formation and involved in the positioning of activated B cells within the follicular microenvironment.

### Impaired B Cell Development in the Neonatal Spleen and Liver and Fetal Liver of DKO Mice

**Figure 4:**
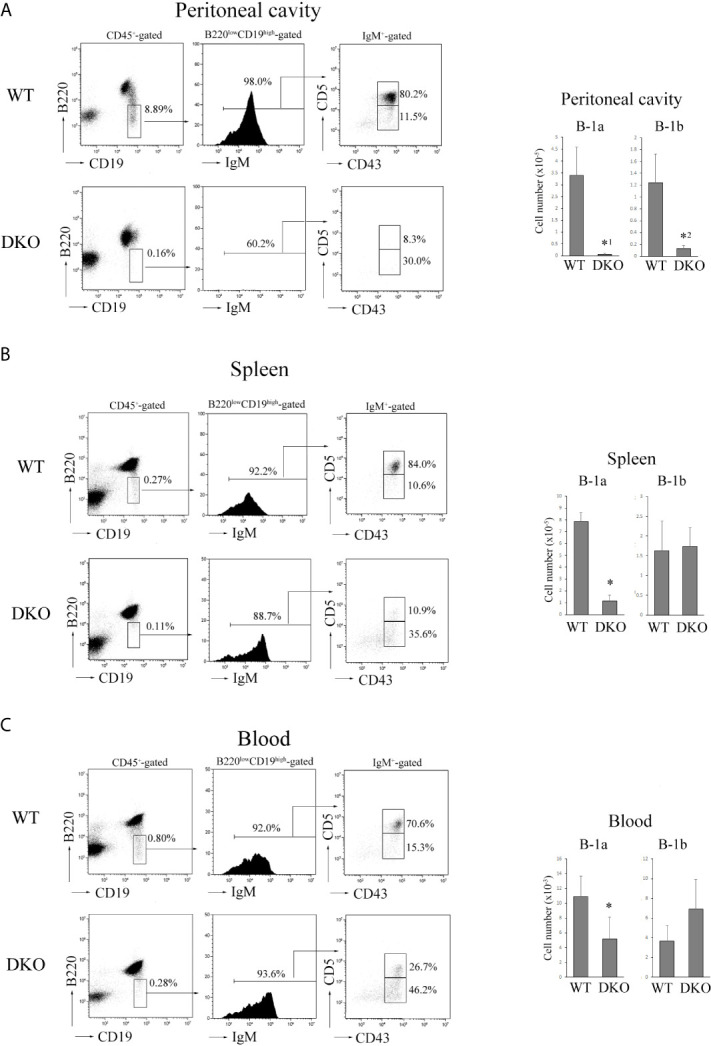
Severe reduction of B-1a cells in the peritoneal cavity, spleen and blood of DKO mice. **(A)** (Left) B220 and CD19 profiles of CD45^+^-gated cells (left), IgM profiles of B220^low^CD19^high^ -gated cells (center) and CD5 and CD43 profiles of IgM^+^-gated cells (right) from the peritoneal cavity of adult WT or DKO mice. The numbers indicate the percentages of B220^low^ CD19^high^ cells, IgM^+^ cells and CD5^+^CD43^+^ (B-1a) or CD5^-^CD43^+^ (B-1b) cells. (Right) The numbers of B-1a and B-1b cells from the peritoneal cavities of WT and DKO mice. The mean values and standard errors are shown (*n*=7). *^1^ *p* < 0.02, *^2^ *p* < 0.03, compared with WT cells. **(B)** (Left) B220 and CD19 profiles of CD45^+^-gated cells (left), IgM profiles of B220^low^CD19^high^ -gated cells (center) and CD5 and CD43 profiles of IgM^+^-gated cells (right) from the spleens of adult WT or DKO mice. The numbers indicate the percentages of B220^low^ CD19^high^ cells, IgM^+^ cells and CD5^+^CD43^+^ (B-1a) or CD5^-^CD43^+^ (B-1b) cells. (Right) The numbers of B-1a and B-1b cells from the spleens of WT and DKO mice. The mean values and standard errors are shown (*n*=7). *p < 0.02, compared with WT cells. **(C)** (Left) B220 and CD19 profiles of CD45^+^-gated cells (left), IgM profiles of B220^low^CD19^high^ -gated cells (center) and CD5 and CD43 profiles of IgM^+^-gated cells (right) from the blood of adult WT or DKO mice. The numbers indicate the percentages of B220^low^ CD19^high^ cells, IgM^+^ cells and CD5^+^CD43^+^ (B-1a) or CD5^-^CD43^+^ (B-1b) cells. (Right) The numbers of B-1a and B-1b cells from the blood of WT and DKO mice. The mean values and standard errors are shown (*n*=5). *p < 0.02, compared with WT cells.

Both B-1a (B220^low^CD19^high^IgM^+^CD43^+^CD5^+^) and B-1b (B220^low^CD19^high^IgM^+^CD43^+^CD5^-^) cells were almost absent in the peritoneal cavity of adult DKO mice (8-12 weeks of age) (**Figure 4A**). Although B-1b cells were present in the spleen and blood of adult DKO mice, B-1a cells were significantly decreased there (**Figures 4B, C**). These data indicated that B-1a cells were not only reduced in the peritoneal cavity, but also in the blood and spleen of DKO mice.

**Figure 5:**
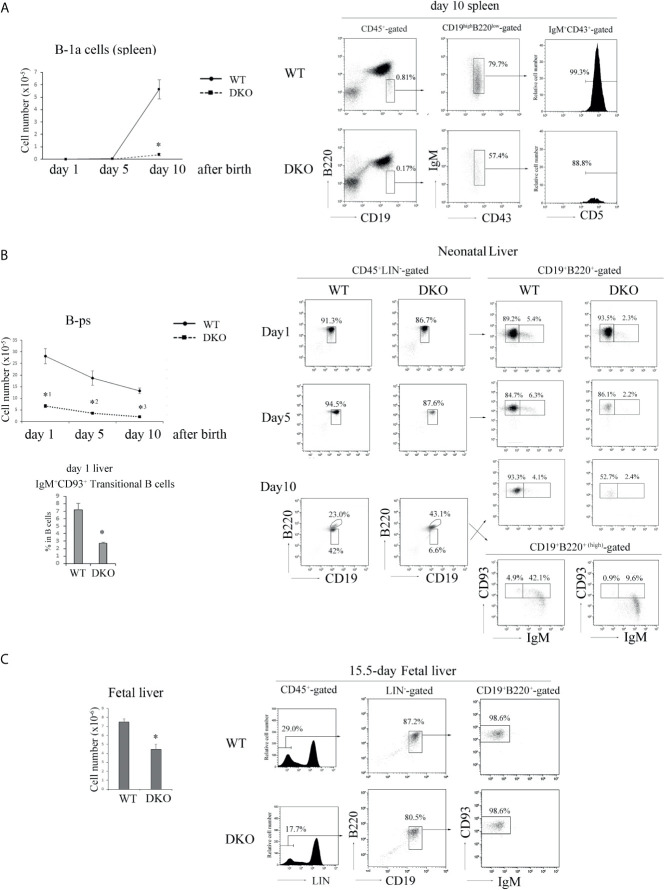
Impaired B cell development in the neonate and fetus periods of DKO mice. **(A)** (Left) The number of B-1a (B220^low^ CD19^high^IgM^+^CD43^+^CD5^+^) cells in day 1, 5 and 10 neonatal spleens of WT and DKO mice (*n*=5-15). *p < 0.001, compared with WT mice. (Right) B220 and CD19 profiles of CD45^+^-gated cells (left), IgM and CD43 profiles of B220^low^CD19^high^-gated cells (center) and CD5 profiles of IgM^+^CD43^+^-gated cells (right) from day 10 neonatal spleens of WT and DKO mice. The numbers indicate the percentages of B220^low^ CD19^high^ cells, IgM^+^CD43^+^ cells and CD5^+^ cells. **(B)** (Left upper) The number of B-ps (CD45^+^LIN^-^CD19^+^ B220^+^CD93^+^IgM^-^) in day 1, 5 and 10 neonatal livers of WT and DKO mice (*n*=5). *^1^ *p* < 0.001, *^2^ *p* < 0.003, *^3^ *p*<0.001, compared with WT mice. (Left lower) Ratios of transitional B cells (CD45^+^CD19^+^B220^+^IgM^+^ CD93^+^) in day 1 neonatal livers of WT and DKO mice. *p < 0.008, compared with WT mice. (Right) B220 and CD19 profiles of CD45^+^ LIN^–^gated cells (left), CD93 and IgM profiles of CD19^+^B220^+^ or CD19^+^B220^+(high)^ -gated cells from the livers of day 1, 5 and 10 neonatal WT and DKO mice (right). The numbers indicate the percentages of CD19^+^B220^+^, CD19^+^B220^+(high)^, IgM^-^ CD93^+^ or IgM^+^ CD93^+^ B cells. **(C)** (Left) The number of B-ps in fetal livers of WT and DKO mice (*n*=7). *p < 0.001, compared with WT mice. (Right) LIN (CD3, CD4, CD8, CD11b, Gr-1, NK1.1, TER119) profiles of CD45^+^-gated cells (left), B220 and CD19 profiles of LIN^–^gated cells (center), CD93 and IgM profiles of CD19^+^B220^+^-gated cells (right) in fetal livers of WT and DKO mice. The numbers indicate the percentages of LIN^-^ cells, CD19^+^ B220^+^ cells and CD93^+^IgM^-^ cells.

Since most B-1 cells are appeared at the neonatal stage (23), we examined B-1 cell development in neonatal spleens and livers. B-1a cells were clearly detected at day 10 after birth in the neonatal spleen of WT mice, but the number of B-1a cells in the neonatal spleen of 10-day-old DKO mice was only a one-tenth of that of WT mice (**Figure 5A**).

There are substantial controversies about precise progenitors of B-1 cells and ontogenetic relationships between cells designated (25, 42–44) as B-1 progenitors. B220^+^CD43^+^ cells in fetal liver (42), which may overlap with CD19^+^B220^+^CD93^+^ cells, and CD19^+^B220^+^CD93^+^ IgM^+^ transitional B cells (TrB) in neonatal spleen (23, 25) were reported to include the progenitor/precursors having the ability to differentiate into B-1 cells. Notwithstanding some disagreement, here we will refer to these reported phenotypes as those of B-ps and TrB. We examined their numbers and proportions in the livers of 1-, 5-, and 10-day-old neonatal DKO mice. B-ps (CD45^+^LIN^-^CD19^+^B220^+^CD93^+^IgM^-^) were less than 20% of those of the livers of neonatal WT mice (**Figure 5B**). The proportion of IgM^+^CD93^+^ transitional B cells was also significantly decreased in the neonatal liver of 1-day-old DKO mice (**Figure 5B**). IgM^+^CD93^-^ B cells which demonstrated higher expression of B220 than that of IgM^-/+^CD93^+^ B cells appeared in the liver of 10-day-old mice, and might be mature B cells derived from the bone marrow in DKO mice (**Figure 5B**).

Subsequently, we examined the number of B-ps in E15.5 fetal livers. As shown in **Figure 5C**, the number of B-ps in the livers of E15.5 fetal DKO mice was decreased by more than 40% of that of fetal WT mice. These data indicate that Rap1 is necessary for survival, proliferation or differentiation of B-ps in neonatal and fetal livers.

### Rap1 Is Important for the Contact of B-ps With ALCAM^high^ Stromal Cells *via* CXCL12- and VLA-4/VCAM-1-Dependent Migration

**Figure 6:**
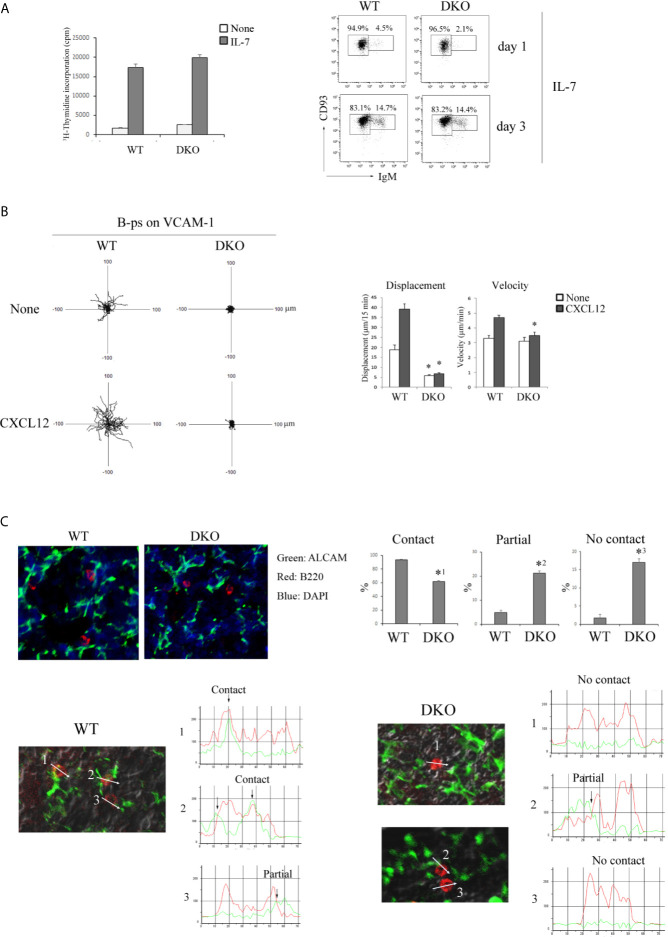
Migration of B-ps on the VCAM-1 is dependent on Rap1. **(A)** (Left) [^3^H]-thymidine uptake by B-ps (CD45^+^LIN^-^ CD19^+^B220^+^CD93^+^IgM^-^). B-ps from the fetal livers of WT and DKO mice were cultured in the absence or presence of IL-7. [^3^H]-thymidine uptake was measured 2 days after the stimulation. The mean values and standard errors are shown. (Right) CD93 and IgM profiles of B-ps which were cultured with IL-7 for 1 and 3 days. The numbers indicate the percentages of IgM^-^ CD93^+^ B-ps or IgM^+^ CD93^+^ transitional B cells. **(B)** (Left) The tracks of WT and DKO B-ps on the VCAM-1 in the absence or presence of CXCL12 are shown. Each line represents a single cell track. (Right) Displacement and velocity of WT and DKO B-ps were measured on the VCAM-1 with or without CXCL12 (*n*=30). *p < 0.001, compared with WT B-ps. **(C)** (Top left) Fetal liver sections of WT and DKO mice were stained with anti-ALCAM (green), anti-B220 (red) and DAPI (blue). (Top right) Proportions of B-p showing contact, partial contact and no contact between B-ps and ALCAM^+^ stromal cells (*n*=30). *^1^ *p* < 0.003, *^2^ *p* < 0.007, *^3^ *p* < 0.004, compared with WT B-ps. (Bottom) Line profiles of B220 and ALCAM intensities are generated along the direction of the arrow. The case where B220 and ALCAM overlapped in the point of more than 80% of each peak intensity, is categorized to ‘contact’; the case where B220 and ALCAM overlapped in the point of more than 30% of each peak intensity, is categorized to ‘partial’; the case where B220 and ALCAM overlapped in the point of less than 30% of each peak intensity, is categorized to ‘no contact’.

B-cell development in the fetal liver is supported by IL-7 (31). B-ps (CD45^+^LIN^-^ CD19^+^B220^+^CD93^+^IgM^-^) were purified from fetal livers of E15-15.5 WT and DKO mice by cell sorting (**Figure S2A** in **Supplementary Material**) and cultured in the presence of IL-7. As shown in **Figure 6A**, the proliferative response of Rap1-deficient B-ps to IL-7 was similar to that of WT B-ps. The frequency of differentiation of Rap1-deficient B-ps into transitional B cells (B220^+^CD19^+^CD93^+^IgM^+^) was also similar to that of WT B-ps (**Figure 6A**). In addition, there was no difference in the expression of the IL-7 receptor between WT and DKO B-ps (**Figure S2B** in **Supplementary Material**). These data indicate that Rap1 is not involved in IL-7-dependent proliferation and differentiation of B-ps.

We explored the effects of Rap1 on VLA-4/VCAM-1-dependent migration of B-ps in the presence or absence of CXCL12. As shown in **Figure 6B**, WT B-ps, but not Rap1-deficient B-ps, actively migrated on the VCAM-1-coated plate. In particular, the displacement of Rap1-deficient B-ps was markedly impaired regardless of CXCL12. The expression of CXCR4 and VLA-4 in DKO B-ps was similar to those of WT B-ps (**Figure S2C** in **Supplementary Material**). These results indicated that the locomotion of B-ps in the fetal liver was Rap1-dependent.

ALCAM^+^ stromal cells, which express VCAM-1 and produce CXCL12, chemoattract B-ps (31). B-ps survive and proliferate when in contact with ALCAM^+^ stromal cells in the fetal liver (31). By immunostaining of fetal livers, we examined the contacts between B-ps and ALCAM^+^ stromal cells. As shown in **Figures 6C** and **S2D** in **Supplementary Material**, the proportion of B-ps that demonstrated ‘contact’ with ALCAM^+^ stromal cells was significantly lower in the livers of fetal DKO mice than those of fetal WT mice.

These results suggest that the reduction in the number of B-ps might be caused by the impairments in their interaction with IL-7-producing stromal cells in the fetal liver of DKO mice.

## Discussion

We propose from the data presented that the absence of Rap1-deficient B cells in LN occurs as a result of defective homing or retention, leading to localization of B cells in the blood and spleen, which were consistent with the previous papers (11, 12). In contrast, B-1a cells are markedly reduced even in the spleen and blood of adult DKO mice, and the number of B-ps was diminished in neonatal and fetal livers. Although Rap1 was reported to be directly involved in intracellular signaling to induce B-1 development using adult BM cells (28, 29), there were no defects in the proliferation and differentiation of Rap1-deficient B-ps from fetal liver in response to IL-7 *in vitro*, indicating that Rap1 is dispensable for IL-7-dependent development of B-ps. On the other hand, Rap1-deficient B-ps barely migrated on the VCAM-1 in the presence of CXCL12. Previous papers have reported that ALCAM^high^ non-hematopoietic cells were found to express VCAM-1 and support hematopoiesis by producing chemokines such as CXCL12 and cytokines, in particular, IL-7 (30–32). The contacts of B-ps with IL-7-producing stromal cells were impaired in the fetal liver of DKO mice. Defective CXCL12-dependent positioning of Rap1-deficient B-ps might have decreased their encounters and interaction with IL-7-producing stromal cells in the fetal liver. It is unproven but here is why we interpret the findings as suggesting a causal relationship between defective locomotion and reduced number of B-ps in fetal liver of DKO mice.

On the other hand, B-1b cells were absent in the peritoneal cavity, but present in the blood and spleen of adult DKO mice. These data indicate that Rap1 is necessary for the positioning of B-1b cells into the peritoneal cavity, but might be dispensable for differentiation of B-1b cells by B-cell lymphopoiesis in the bone marrow.

Previous paper (11) demonstrated that lack of Rap1b reduced the number of pro/pre-B cells and immature B cell in bone marrow. In another previous paper (12) and this study, the number of pro/pre-B cells was slightly, but not significantly reduced in bone marrow. The numbers of pro/pre-B cells were varied between individuals because Rap1-deficiency possibly might affect the differentiation of pre-B cells to immature B cells. Rap1a null mice did not show any obvious defects in the differentiation and maturation of lymphoid cells (45, 46). Taken together, Rap1b might be involved in maximal B cell development in bone marrow. Furthermore, bone marrow-derived Rap1b-deficient pro/pre-B cells normally proliferated in the presence of IL-7, but their adhesion to stromal cell line was reduced compared with WT cells (11), suggesting that Rap1b might play important roles in the interaction of pro/pre-B cells with IL-7-producing stromal cells in bone marrow. As previously reported (43), fetal pro-B cells mainly differentiated into B-1 cells, but the adult pro-B cells mainly differentiated into B-2 cells. Rap1 might be more indispensable for the interaction of B-ps with stromal cells in fetal liver than in adult bone marrow, because the steady-state of B-1a cells was severely impaired by Rap1-deficiency.

The recirculating mature B cells in bone marrow were not reduced in Rap1b null mice (11, 12), while the percentages of mature B cells in bone marrow of DKO mice were reduced to approximately one-fourth of WT mice (**Figure 1F**), suggesting that Rap1a and Rap1b have redundant roles in the repopulation of mature B cells in bone marrow. In contrast to DKO mice, B-1a and b cells normally exited in the peritoneal cavity of Rap1b null mice (12). Rap1a null mice showed normal serum level of IgM (45). These data suggest that Rap1a and Rap1b have redundant roles in B-1 development and positioning in the peritoneal cavity.

In this study, we revealed that the development of GCs in B-cell follicles during T cell-mediated immune responses was Rap1-dependent. Upon encountering antigens, activated B cells undergo multiple migratory steps, which are dependent on chemoattractants such as 7α,25-OHC and CCL21, that are expressed in distinct stromal cells (13, 14, 47). The upregulation of EBI2 and CCR7 expression on antigen-activated B cells results in their movement towards outer follicular regions and the T-cell zone to seek antigens and the help of T cells. Since antigen-activated B cells maintain CXCR5 expression, subsequent down-regulation of EBI2 and CCR7 expression induces their migration towards the central FDC-dense areas where they proliferate and form GCs (13, 47). We found that Rap1-deficiency reduced the directional locomotion of the activated B cells along the gradient of 7α,25-OHC *in vitro*, which may partly delay their proliferative responses to antigens and the formation of GCs. In addition, the movement of Rap1-deficient B-cell blasts on VCAM-1 along a CXCL12 gradient was also impaired. In the GCs, CXCL12 is expressed in more abundantly in the dark zone than in the light zone, and is required for the segregation of the dark and light zones (15, 17). Rap1 might possibly influence the locomotion of CXCR4-expressing centroblast to the dark zones.

BCR-mediated signaling and internalization of antigens are critical for the differentiation of B cells into antibody-producing cells (48, 49). B cells recognize antigens on the antigen-presenting cells (APCs) such as follicular dendritic cells, subcapsular sinus macrophages and marginal zone B cells through the B cell receptor (BCR) (49, 50). B cells capture BCR-bound antigens from APC and delivered to major histocompatibility complex (MHC) II-containing vesicles *via* actin- and microtubule-dependent processes (51–53). Peptide –MHC-II complexes are presented to T cells, which provide the signals required for B cell activation. Thus, B-cell differentiation into plasma cells is dependent on the B cell-APC interaction and BCR-dependent cytoskeletal reorganization (51–55). Rap1 plays central roles in integrin-dependent adhesion (4, 9, 10) and BCR-induced reorganization of actin and MTOC polarization (56, 57). Therefore, Rap1-deficiency might impair B-cell contact with APC and BCR-dependent antigen internalization, which also contributes to defective development of GC.

Our previous study demonstrated that Rap1 guanine nucleotide exchange factors, Ras/Rap association-guanine nucleotide exchange factor (RA-GEF)-1 and 2 play critical roles in the retention of immature B cells in the BM (40). This study confirms that Rap1 activation is required for immature B-cell retention in the BM. In contrast to T cells, immature B cells egress passively from the BM, independent of pertussis toxin (PTX)-sensitive GPCR signaling, such as that of sphingosine-1-phospohate (S1P) (41). On the other hand, the retention of these cells in the BM strictly depends on amoeboid motility mediated by CXCR4 and VLA-4 (41). In addition, RA-GEF-1 and 2 are dispensable for naïve B-cell homing into peripheral LNs (40). However, in this study, the deficiency of Rap1 in B cells was found to be indispensable for B-cell homing into LNs. Therefore, other Rap1GEFs, such as C3G, may compensate for the chemokine-dependent integrin activation of naïve B cells required for transmigration through the HEVs.

Various aspects of B-cell development rely on chemokine- and integrin-dependent adhesion and migration, in which Rap1 plays central roles. Hence, it is important to clarify the regulatory mechanisms of Rap1 activation and downstream effector molecules is important to understand B-cell proliferation, differentiation, and function.

## Data Availability Statement

The raw data supporting the conclusions of this article will be made available by the authors.

## Ethics Statement

The animal study was reviewed and approved by Regulations for the Care and Use of Laboratory Animals in Kitasato University.

## Author Contributions

SI and KK designed, performed experiments, and wrote the paper. TS, RiS, RM and HS performed the experiments. RyS, HF, AN, SS and AI contributed to the preparation of essential materials and commented on the experiments and paper. All authors contributed to the article and approved the submitted version.

## Conflict of Interest

The authors declare that the research was conducted in the absence of any commercial or financial relationships that could be construed as a potential conflict of interest.

## Abbreviations

GC, germinal center; NP-CGG, nitrophenyl-chicken gamma globulin; IL-7, interleukin-7; CXCL12, C-X-C motif chemokine 12; VCAM-1, vascular cell adhesion molecule-1; ALCAM, activated leukocyte cell adhesion molecule.
