# Supplementary figures and images for: Rap1 Is Essential for B-Cell Locomotion, Germinal Center Formation and Normal B-1a Cell Population
Source: Front Immunol. 2021 Jun 1;12:624419. doi: 10.3389/fimmu.2021.624419 (PMC8203927; doi:10.3389/fimmu.2021.624419)

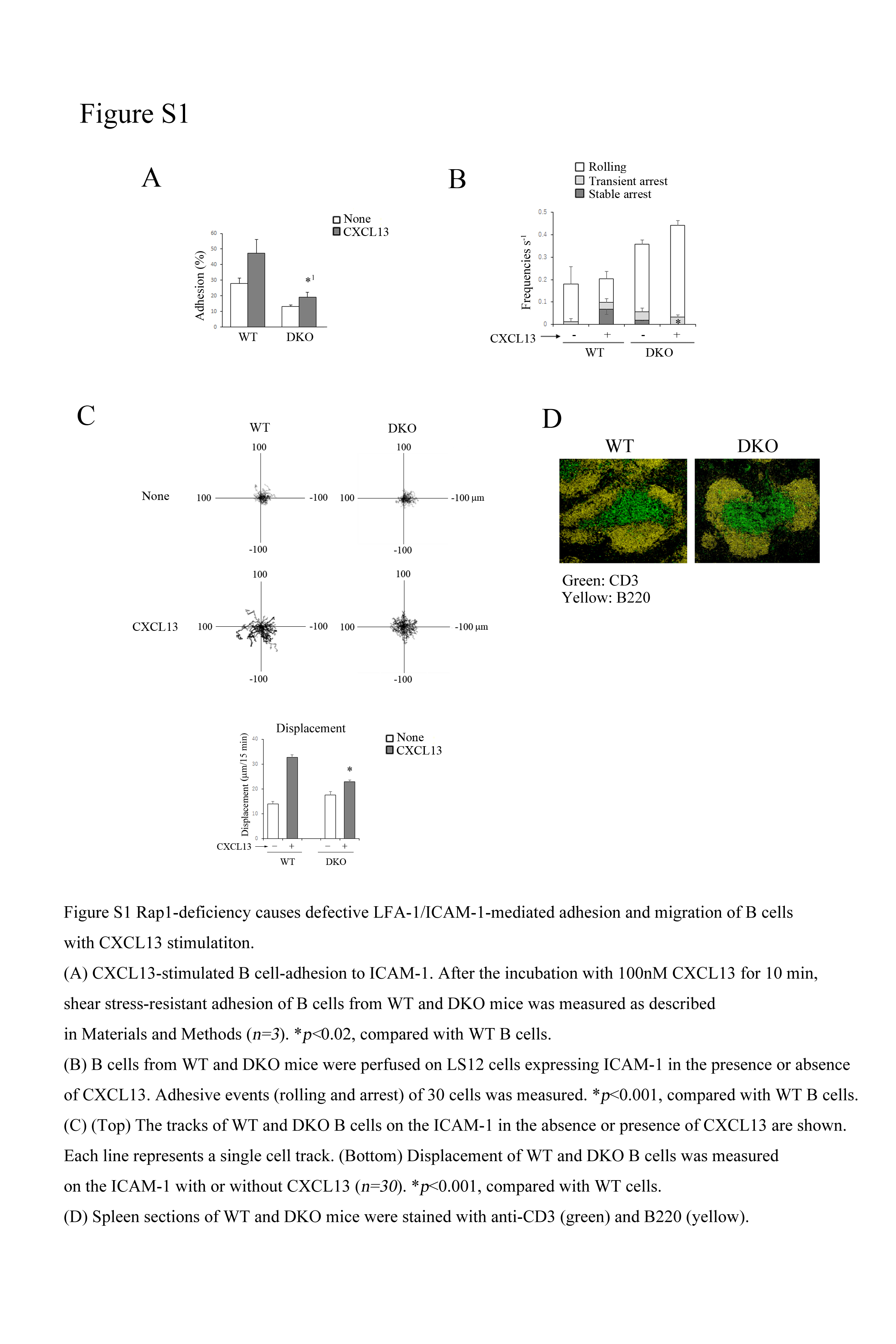

Supplement: Supplementary file 1 [file Image_1.jpg]

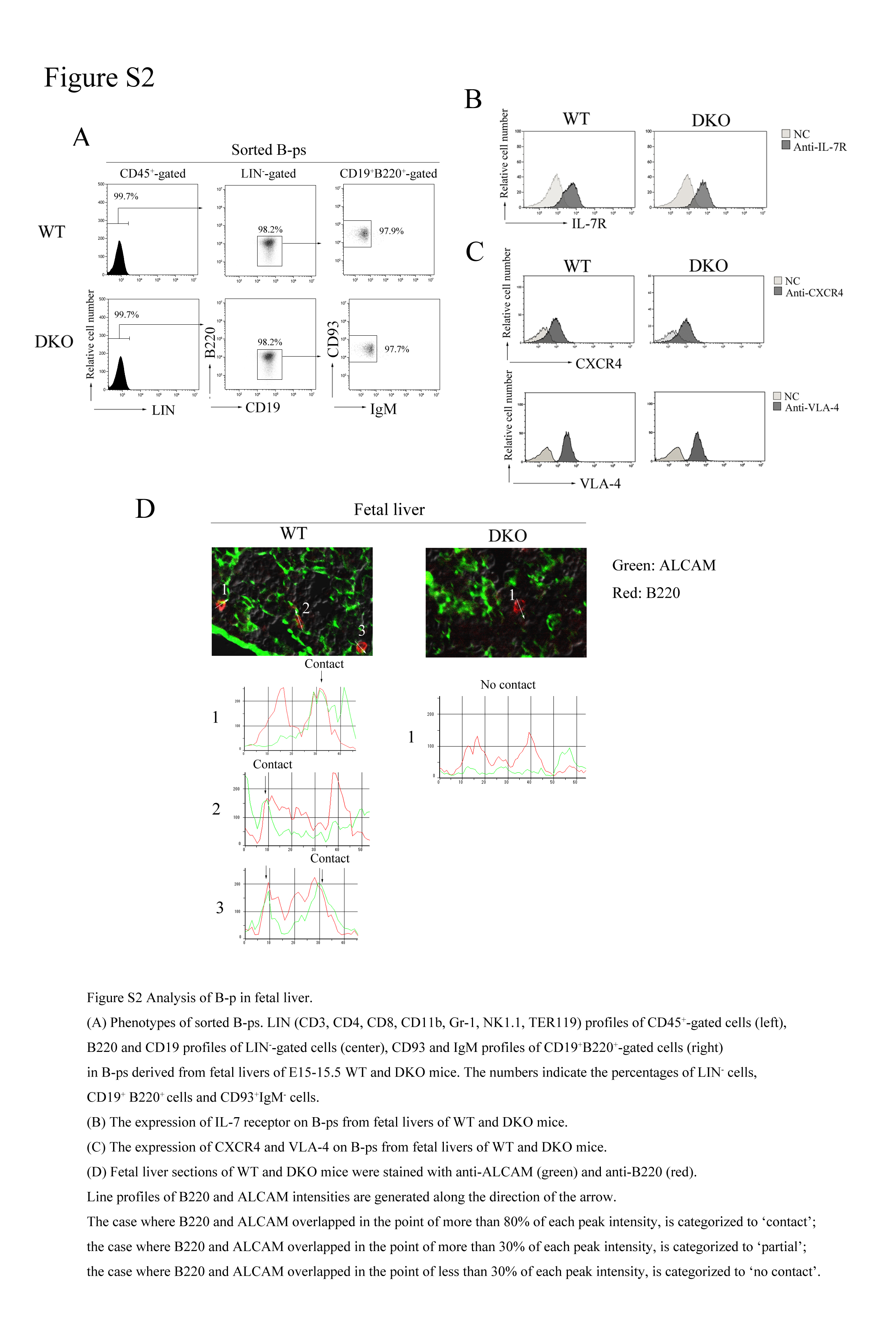

Supplement: Supplementary file 2 [file Image_2.jpeg]
